# Robertsonian Translocation between Human Chromosomes 21 and 22, Inherited across Three Generations, without Any Phenotypic Effect

**DOI:** 10.3390/genes15060722

**Published:** 2024-06-01

**Authors:** Concetta Federico, Desiree Brancato, Francesca Bruno, Daiana Galvano, Mariella Caruso, Salvatore Saccone

**Affiliations:** 1 Department of Biological, Geological and Environmental Sciences, University of Catania, Via Androne 81, 95124 Catania, Italy; concetta.federico@unict.it (C.F.); desiree.brancato@phd.unict.it (D.B.); francesca.bruno@unict.it (F.B.); 2 Cytogenetic Laboratory, A.O.U. Policlinico Vittorio Emanuele, 95124 Catania, Italy; daianagalvano@gmail.com (D.G.); mariella.caruso55@gmail.com (M.C.)

**Keywords:** human karyotype, Robertsonian translocation, fluorescence in situ hybridization, chromosome 21, chromosome 22, karyotype evolution, nuclear position effect, modes of chromosome evolution

## Abstract

Chromosomal translocations can result in phenotypic effects of varying severity, depending on the position of the breakpoints and the rearrangement of genes within the interphase nucleus of the translocated chromosome regions. Balanced translocations are often asymptomatic phenotypically and are typically detected due to a decrease in fertility resulting from issues during meiosis. Robertsonian translocations are among the most common chromosomal abnormalities, often asymptomatic, and can persist in the population as a normal polymorphism. We serendipitously discovered a Robertsonian translocation between chromosome 21 and chromosome 22, which is inherited across three generations without any phenotypic effect, notably only in females. In situ hybridization with alpha-satellite DNAs revealed the presence of both centromeric sequences in the translocated chromosome. The reciprocal translocation resulted in a partial deletion of the short arm of both chromosomes 21, and 22, with the *ribosomal RNA* genes remaining present in the middle part of the new metacentric chromosome. The rearrangement did not cause alterations to the long arm. The spread of an asymptomatic heterozygous chromosomal polymorphism in a population can lead to mating between heterozygous individuals, potentially resulting in offspring with a homozygous chromosomal configuration for the anomaly they carry. This new karyotype may not produce phenotypic effects in the individual who presents it. The frequency of karyotypes with chromosomal rearrangements in asymptomatic heterozygous form in human populations is likely underestimated, and molecular karyotype by array Comparative Genomic Hybridization (array-CGH) analysis does not allow for the identification of this type of chromosomal anomaly, making classical cytogenetic analysis the preferred method for obtaining clear results on a karyotype carrying a balanced rearrangement.

## 1. Introduction

Animal species are normally characterized by a karyotype with a constant number of chromosomes across all individuals, and closely related species may exhibit similar or notably different karyotypes. For example, the Indian muntjac (*Muntiacus muntjak*), belonging to the Artiodactyla, has a karyotype of only seven chromosomes in males and six in females, which is markedly distinct from that of the Chinese muntjac (*Muntiacus reevesi*), a closely related species with 23 pairs of chromosomes [1,2,3]. Similarly, in goats (*Capra hircus*) and sheep (*Ovis aries*), there are differences in their karyotypes due to the presence of three pairs of metacentric chromosomes in sheep that are absent in goats, obtained by Robertsonian translocations between acrocentric chromosomes [4,5]. In marsupials, species within the family Dasyuridae exhibit a high degree of karyotype conservation, while those within the family Macropodidae display significantly different karyotypes [6].

In birds, the karyotype is typically highly conserved, characterized by a high number of microchromosomes alongside some macrochromosomes [7,8]. However, an exception to this conservation is observed in diurnal raptors, where both macrochromosomes and microchromosomes are absent [9,10,11]. Reptiles exhibit a notable variability in karyotype composition and microchromosome number, with species ranging from a diploid number of 20 to 68 and displaying karyotypes with up to 56 microchromosomes [12]. Similarly, in amphibians, there is a wide range of karyotypic variation, with some species sharing very similar karyotypes while others display significant differences in both number and morphology. This diversity is particularly evident, for example, among salamanders [13]. Within the Erythrinidae family of fish, karyotype diversity is considerable, with species exhibiting a range of 2n configurations from 39 to 54 chromosomes [14,15]. However, it is worth noting that species belonging to the genus *Hoplias* within this family maintain a constant number of chromosomes with 2n = 50 [16].

Within the order of primates, humans are closely related to anthropoid apes, such as gorillas (*Gorilla gorilla*), chimpanzees (*Pan paniscus*), and orangutans (*Pongo pygmaeus*). These species, along with the cercopithecoid group, which includes the rhesus macaque (*Macaca mulatta*), belong to the Catarrhines or Old World monkeys. The Platyrrhines, on the other hand, include the New World monkeys. Across primates, karyotypes typically consist of around 40–50 chromosomes with various morphologies [17]. The human karyotype consists of 46 chromosomes of diverse sizes and shapes, including five pairs of acrocentric chromosomes. In species more closely related to humans, many chromosomes are highly conserved compared to their human counterparts. However, as we trace back to the common primate ancestor, homologous chromosomes become less recognizable compared to those in humans [18,19,20]. One notable difference between the human karyotype and that of great apes is the presence of a large submetacentric chromosome in humans, which is absent in other anthropoid primates. This chromosome results from a Robertsonian fusion that reduced the human karyotype by one chromosome pair compared to the ancestral karyotype of the great apes [21,22]. Additionally, the evolutionary history of each human chromosome, such as chromosome 7, has been reconstructed, revealing various events like insertions, inversions, translocations, and breakages [23]. Therefore, chromosomes can evolve through a wide range of rearrangements, including not only Robertsonian translocations but also translocations, inversions, and breakages.

Chromosomal rearrangements in humans are often linked to various pathologies or syndromes. One of the most common and well-known chromosomal syndromes is Down syndrome, characterized by a karyotype of 47 chromosomes with three copies of chromosome 21. This extra chromosome 21 can be present autonomously or be associated with another acrocentric chromosome in a Robertsonian translocation, such as chromosome 14 or chromosome 21 itself, in translocations 14;21 or 21;21, respectively [24,25]. In other cases, chromosomal rearrangements can lead to tumor-related pathologies. For instance, the classical translocation between chromosome 8 and chromosome 14 associated with Burkitt’s lymphoma positions the *c-myc* gene downstream of the immunoglobulin promoter site, leading to its activation [26,27]. Another example is the translocation between chromosome 9 and chromosome 22, resulting in the formation of the Philadelphia chromosome, which is associated with the development of leukemia. This translocation leads to the fusion of the *breakpoint cluster region* (*bcr*) and *Abelson* (*abl*) genes, forming the chimeric gene *bcr-abl*, which plays a role in leukemia pathogenesis [28].

There are also chromosomal rearrangements associated with genetic pathologies that do not involve mutated genes, such as the translocation between chromosomes 7 and 12 associated with childhood acute myeloid leukemia [29]. In this translocation, there are no mutations in the genes located at the breakpoint sites. Instead, the *Motor Neuron and Pancreas Homeobox 1* (*MNX1*) gene (previously known as *HLXB9*) is repositioned within the nucleus due to the chromosomal translocation, leading to its activation [30,31,32]. Similarly, in a translocation between chromosome 14 and chromosome 18, the *B-cell lymphoma 2* (*bcl2*) gene is repositioned, resulting in its activation [33]. Therefore, the presence of a classical gene mutation is not always necessary for a significant phenotypic effect, such as the onset of a genetic pathology. The repositioning of a gene within the nucleus is sufficient to modify its transcriptional features and contribute to the development of the pathology [34].

The preservation of evolutionary chromosomal rearrangements over time involves several factors. One key aspect is the absence of detrimental phenotypic effects associated with the rearrangement. Evolutionary success relies on the ability of organisms to adapt to their environment and reproduce, so any chromosomal rearrangement that negatively impacts these factors is less likely to be preserved. Comparative analysis of the positioning of human loci and their homologs in primates, which have experienced chromosomal rearrangements such as inversions, translocations, or other alterations, has revealed intriguing findings. Despite differences in their positions on metaphase chromosomes, these studies have shown that the arrangement of genes within the interphase nucleus remains consistent, and their transcriptional characteristics remain unaltered [35]. It is important to note that karyotype analysis for diagnostic purposes may also identify changes in chromosome morphology that are not necessarily associated with clinical phenotypic effects. This highlights the complexity of interpreting the relationship between chromosomal rearrangements and their phenotypic consequences [36].

In this study, we show how a Robertsonian translocation between chromosomes 21 and 22 serves as an illustration of a silent chromosomal aberration. Once it occurs in an individual, this translocation can persist within the population randomly, as also detected in other familial members without causing any discernible phenotypic impact.

## 2. Materials and Methods

### 2.1. Preparation of Chromosomes and Nuclei and Chromosomal Banding

Metaphase chromosomes and nuclei were prepared from phytohaemagglutinin (PHA)-stimulated peripheral blood lymphocytes of healthy voluntary subjects according to standard cytogenetic procedures, as described previously [35]. G-banding was performed by a standard procedure using trypsin (Invitrogen, Thermo Fisher Scientific Italia, Milano, Italy) and Giemsa stain (Merck, Darmstadt, Germany).

### 2.2. DNA Probes, In Situ Hybridization, and Detection

The probes used for fluorescence in situ hybridizations (FISH) to detect euchromatic chromosomal bands were Bacterial Artificial Chromosomes (BAC) clones (Table 1). Moreover, to detect the centromeric regions of chromosomes 21 and 22 and the rDNA present in the 21p12 and 22p12 bands, pZ21A [37], P190.22 [38], and RP5-1174A5 [39] probes were used, respectively, kindly provided by M. Rocchi, University of Bari, Italy.

DNA was extracted from the bacteria using commercial kit (Qiagen, Milan, Italy), and the probes were biotin- or digoxigenin-labeled by nick translation (Roche, Mannheim, Germany), and hybridized as previously described [40]. Briefly, hybridization was performed at 37 °C for 16 h. In the case of the BAC probes, a 30 min pre-annealing step with an excess of unlabeled human Cot-1 DNA was carried out. After post-hybridization washes at 60 °C with 0.1xSSC (1xSSC is 0.15 M NaCl, 0.015 M sodium citrate), hybridization detection of the biotin- and digoxigenin-labeled probes was performed using rhodamine-conjugated avidin and fluorescein-conjugated anti-digoxigenin antibody, respectively. The chromosomes and nuclei were stained with 4′,6-diamidin-2-fenilindolo (DAPI). Images were captured using epifluorescence microscopy (Olympus AX70) and a Photometrics cooled CCD camera with MacProbe v4.2 software (Applied Imaging, Newcastle, UK).

### 2.3. Radial Nuclear Location Analysis

The cell nuclei were analyzed for the evaluation of the radial location of the rearranged chromosome. This was obtained using 2D FISH analysis as previously described [40]. Briefly, the nuclei were hybridized with single-locus probes, and at least 200 hybridized nuclei were randomly recorded. The radial nuclear location (RNL) of each hybridization spot, indicated as a ratio of the nuclear radius, was assigned to each hybridization signal, assigning a value of 0 for a signal located in the center, a value of 1 for a signal located at the border of the nucleus, and a value between 0 and 1 for the other signals between the border and the center of the nucleus. The RNL was statistically defined as the median value ± confidence interval of the analyzed hybridization signals. Three probes previously located in the inner (median value < 0.65), intermediate (median value between 0.65 and 0.70), and peripheral parts (median value > 0.70) of the nucleus (Table 1) were used as the control [40].

The statistical analyses were carried out using the software Prism v.8.0 (GraphPad Software, San Diego, CA, USA). The statistical differences between pairs of groups were obtained by Student’s two-tailed *t* test. The levels of significance of the differences between the groups were set at: *p* < 0.05 (*), *p* < 0.01 (**), and *p* < 0.0001 (***).

## 3. Results

### 3.1. Detection of a Karyotype with a Robertsonian Translocation

**Figure 1:**
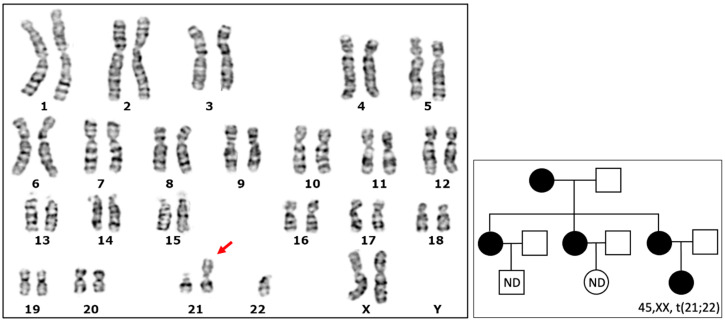
Karyotype with the Robertsonian translocation. **Left**: G-banded karyotype showing the small metacentric chromosome, indicated by the red arrow, resulting from the translocation between chromosomes 21 and 22. **Right:** Genealogical tree showing the transmission of the rearranged chromosome in the present family. □: Male subject; ○: female subject; ●: female carrying t(21;22); nd: subject with karyotype not determined.

We casually identified a karyotype in a healthy 20-year-old female subject that consisted of 45 chromosomes. Notably, this karyotype included a small metacentric chromosome resulting from a translocation between chromosome 21 and chromosome 22 (Figure 1). We expanded the karyotype analysis to other members of the family, revealing the same chromosome configuration, 45,XX,t(21;22), in mother, sister, and daughter (Figure 1). Remarkably, all individuals with this chromosomal configuration were healthy, without any associated phenotypic effects. Furthermore, there were no apparent issues with their gamete vitality, and their fertility appeared to be unaffected.

### 3.2. Genomic Features of the t(21:22) Chromosome

The rearranged chromosome t(21;22) underwent analysis to determine whether it exhibited an altered genetic configuration compared to the two originating chromosomes. FISHs were performed using probes located at different positions along chromosome 21 and chromosome 22, including centromeric and ribosomal RNA sequences. The telomeric end of the long arm of chromosome 21 was examined using the BAC probes RP11-729O4 and RP11-135B7, while those of chromosome 22 were examined with the BAC probes RP11-164E23 and RP11-931F19. The probes within the q arms of chromosomes 21 and 22 were RP11-345K23 and RP11-112D4, respectively (see Table 1). Additionally, the centromeric regions of chromosomes 21 and 22 were identified using the probes pZ21A and p190.22, respectively, and the rDNA region was targeted using the PAC probe RP5-1174A5.

**Figure 2:**
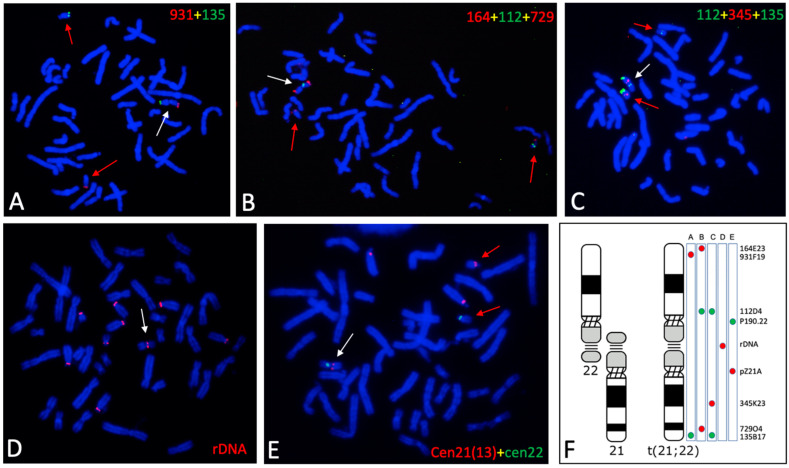
Genetic features of the translocated chromosome. The images show the locations of different probes from chromosomes 21 and 22. The probes are indicated inside each image with the fluorochrome used for detection: rhodamine (red) or fluorescein (green). The chromosomes were stained with DAPI. (**A**) 931: RP11-931F19; 135: RP11-135B17; (**B**) 164: RP11-164E23; 112: RP11-112D4; 729: RP11-729O4; (**C**) 112: RP11-112D4; 345 RP11-345K23; 135: RP11-135B17; (**D**) rDNA: RP5-1174A5; (**E**) cen21(13): pZ21A; cen22: P190.22. In the case of pZ21A, hybridization was observed also in chromosome 13, clearly distinguishable from chromosome 21, the former being larger. (**F**) ideograms of the two normal chromosomes 21 and 22 and the ideograms of the rearranged chromosome, as determined by the FISH results. To the right of the t(21;22) ideogram, the pairs of probes are indicated whose results are shown in (**A**–**E**): this indicates their names (RP11 is not indicated), their positions along the chromosome, and the fluorochrome used in the corresponding image. For each FISH, tens of metaphases were observed, and all showed the presence of the rearranged chromosome. The red and white arrows indicate the normal chromosomes 21 and 22 and the translocated t(21;22) chromosome, respectively.

The results obtained from the FISH analyses using various probe combinations (Figure 2) indicate that the q arms of chromosomes 21 and 22, which fused to form the new metacentric t(21;22) chromosome, maintained their normal genetic configuration (Figure 2A–C). Moreover, the rDNA region was clearly detected in the middle part of the t(21;22) chromosome (Figure 2E). Regarding the centromeric sequences of chromosomes 21 and 22, both were observed in the rearranged chromosome, indicating the presence of two distinct centromeric sequences within the same chromosome (Figure 2D). This configuration suggests that the new metacentric chromosome is dicentric, a feature typically associated with chromosome instability during chromatid separation in anaphase.

**Figure 3:**
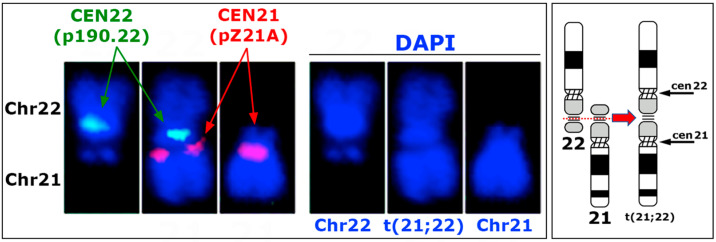
Centromere inactivation in the t(21;22) chromosome. **Left**: Hybridization with the probe specific for the centromeric region of chromosomes 21 (red) and 22 (22). The t(21;22) chromosome shows the typical centromeric signal only with the probe of chromosome 22. In the case of the probe detecting the centromere of chromosome 21, this is clearly split in the two chromatids. Middle: The same chromosomes only stained with DAPI. **Right**: An ideogram of the translocated chromosome showing the absence of the terminal part of the p-arm from both chromosomes 21 and 22.

The centromeric regions of the t(21;22) chromosome were further analyzed through dual-color FISH using probes specific to the centromeric sequences of chromosomes 21 and 22. FISH results detected distinct and contrasting signals on the t(21;22) chromosome compared to the two centromeres. Specifically, the centromeric probe p190.22 appeared as a single spot in the primary constriction of the rearranged chromosome, mirroring the pattern observed in the normal chromosome 22. Conversely, the centromeric probe pZ21A yielded two distinct spots, corresponding to each chromatid derived from chromosome 21 (Figure 3). Notably, no chromosome constriction was observed at the location of this hybridization signal. Therefore, the t(21;22) chromosome possesses only one primary constriction, aligning with that of the original chromosome 22. This observation indicates that the t(21;22) chromosome contains the centromeric sequences of both chromosome 21 and chromosome 22. However, it appears that the functionality of the chromosome 21 centromere is not present, as the two chromatids in this region do not exhibit the typical adhesion of the central domain present in a classical centromere.

### 3.3. Radial Nuclear Positioning of the t(21;22) Chromosome

**Figure 4:**
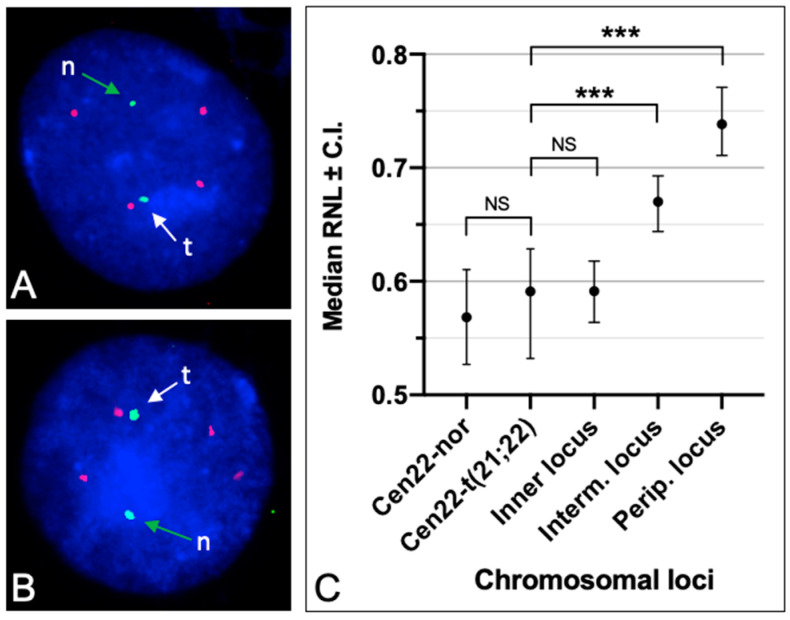
Radial nuclear location (RNL) of the t(21:22) chromosome. The RNL of the t(21;22) chromosome was obtained with the probe P190.22 (centromere of the chromosome 22, two green signals). (**A**,**B**) show two examples of FISH in the cell nuclei to detect the centromere of chromosome 21 and that of 22. The translocated chromosome 22 was highlighted by a very close red signal due to chromosome 21, detected by the pZ21 probe. This is indicated by the white arrow with the letter “t”. The green arrow with the letter “n” indicates the normal chromosome 22. The pZ21 probe detects the centromeres of chromosome 21 and chromosome 13 (four red signals). (**C**) The RNL of the rearranged chromosome t(21;22) was obtained by evaluating the nuclear location of the centromere belonging to chromosome 22. The median value of the detected hybridization signals indicate that the rearranged chromosome (green signals close to a red one) is in the inner part of the nucleus, as for the single chromosome 22. C.I.: 95% confidence interval. Cen22-nor: the normal chromosome 22; Cen22-t(21;22): the rearranged t(21;22) chromosome. Inner locus, Interm. locus, and Perip. locus: loci with known locations in the inner (RP11-213E22), intermediate (RP11-17H7), and peripheral (RP11-79O21) parts of the nucleus, respectively [40]. Statistical differences: NS, and *** indicate not significant and *p* < 0.0001, respectively.

Considering that the chromosomes within nuclei are organized radially based on gene densities, guanine + cytosine (GC)-levels, and the presence of nucleolar organizer regions (NORs) [41], we investigated the radial positioning of the t(21;22) chromosome to determine if it differs or resembles the original chromosomes 21 and 22. To assess this, we determined the RNL of the centromeric region of chromosome 22; it was possible to distinguish signals from the centromere belonging to the individual chromosome 22 and the one associated with the translocated chromosome. This was achieved through dual-color FISH, using probes P190.22 and pZ21A to highlight the two centromeres with different colors (Figure 4A,B). The RNL of the P190.22 probe showed a position in the inner part of the nucleus for the normal chromosome 22, as well as for the derivative chromosome t(21;22). Indeed, the median values of these RNLs were 0.568 and 0.591, respectively, and these were not statistically different (Figure 4C), indicating that the centromeric region of the rearranged chromosome is located within the inner compartment of the nucleus. This observation was further confirmed by comparing the RNL with other control loci (from chromosome 7) situated at the nuclear periphery, in the intermediate nuclear region, or in the inner compartment, where the RNL values were statistically very different (*p* < 0.0001) in the former two cases, and not statistically different in the third case (Figure 4C).

## 4. Discussion

### 4.1. Chromosomes and Genes in the Interphase Nucleus

Chromosomal bands, along with the genes they contain, are not randomly positioned within cell nuclei; rather, they are distributed based on gene density and gene expression patterns [42,43]. In the peripheral region of the nucleus, DNA is highly compacted, and genes in this compartment are typically inactive. Conversely, DNA located in the innermost part of the nucleus is often less condensed, and genes in these regions can be activated more readily [40,41,44,45]. Many studies have identified a phenomenon known as the “position effect”, wherein genes tend to be expressed according to their radial nuclear position [32,46]. Thus, if a gene occupies a different position within the nucleus than usual, this can lead to the development of genetic diseases.

Human housekeeping genes are located in chromosomal bands with the highest GC content and the highest gene density, situated in the inner part of the nucleus and representing the topologically associating domains (TADs) belonging to the A compartment, as identified by the high-throughput chromosome capture (Hi-C) method [47,48,49]. Conversely, tissue specific genes are located in chromosomal bands with the lowest GC content and the lowest gene density, positioned at the nuclear periphery in the B compartment also identified by Hi-C. Chromosomal rearrangements that do not disrupt genes at the breakpoint site can cause gene repositioning within the nucleus, potentially leading to genetic diseases due to the “position effect” [50,51]. On the contrary, if the rearrangement does not result in gene repositioning within the cell nucleus, the rearrangement can be considered asymptomatic, and the rearranged chromosome may persist incidentally in the population where it arises.

**Figure 5:**
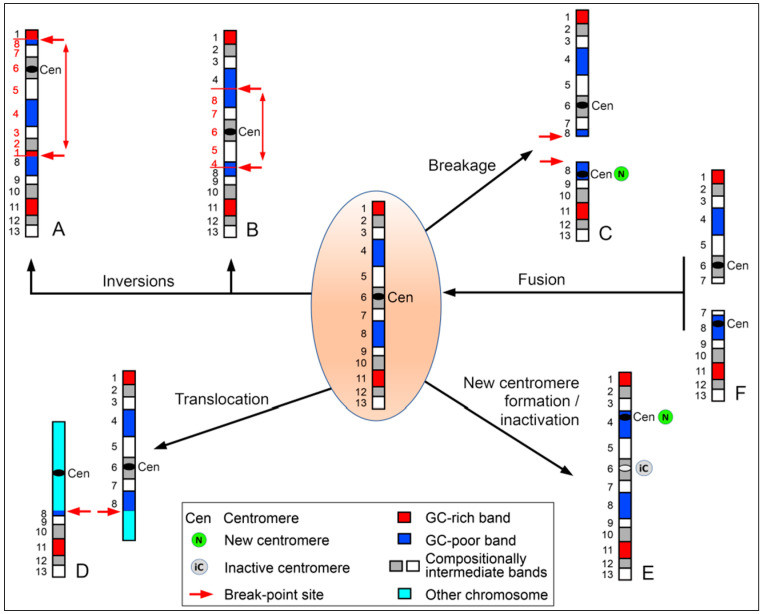
Different modes of chromosome rearrangements and evolutionary success. In the center, the ideogram depicts a standard chromosome featuring GC-rich, GC-poor, and compositionally intermediate bands, together to a canonical centromere. The black arrows indicate various types of rearrangements, resulting in altered organization of chromosomal loci (as numbered on the left). Inversions can lead to the fusion of chromosomal bands with different (**A**) or similar (**B**) GC levels. (**C**) Breakage results in the formation of an acentric chromosome, which may acquire a new centromere, thereby preserving this chromosome segment. (**D**) Reciprocal translocations merge chromosome segments with those of another non-homologous chromosome, juxtaposing chromosomal bands with either similar or different GC levels, as in the case of the inversions. (**E**) A novel centromere may emerge within a euchromatic region, contingent upon the inactivation of the original centromere (iC). (**F**) The fusion of two chromosomes in a Robertsonian-like manner produces a single chromosome that can be faithfully transmitted to daughter cells. To become evolutionarily fixed in the new chromosome, one of the two centromeres will be inactivated over time.

The outcome of chromosomal rearrangements is influenced by the compositional features of the chromosomal regions involved (Figure 5). In one scenario, when the rearrangement involves regions with different GC levels (GC-rich and GC-poor regions), it leads to the repositioning of one of the involved regions within the nucleus. Conversely, in the other scenario where the rearranged regions have similar GC levels, repositioning does not occur since both regions exhibit comparable RNL.

The various modes of chromosome rearrangement and evolution, some of which are summarized above, and the fact that these modifications can occur over time without being highlighted by researchers, are well described in the works of Henry H. Heng [52]. Heng introduced the concept of “*karyotype or chromosomal coding*”, emphasizing the relevance of karyotype dynamics and genomic topology in evolutionary biology. He suggests that the structural organization of chromosomes and the arrangement of genes within chromosomes play critical roles in determining biological outcomes, such as the formation of an altered karyotype, which is often not visible under routine screening [53], as happened in this case, discovered by chance. In this regard, one constraint we previously highlighted in chromosome evolution is the compartmentalization of the genome into subunits with homogeneous features [54], namely chromosomal bands [41,55]. Thus, chromosomes can undergo rearrangements under various inducing stresses [53,56], and the functional unit to consider in the composition and decomposition of new chromosomes should be the chromosomal band [35,40,57], as outlined above (Figure 5).

### 4.2. Chromosomal Rearrangements and Centromere Fate

The translocated t(21;22) chromosome described here harbors both of the original alpha satellite sequences (cen21 and cen22) (Figure 3). Additionally, rDNA was detected in the middle region of the fused chromosomes (Figure 2). This configuration maintains the RNL of the chromosome in the inner part of the nucleus, consistent with the presence of the rDNA. The experimental results by FISH with DNA probes from chromosomes 21 and 22 confirm this preserved radial nuclear location (Figure 4). The fact that carriers exhibit no negative or positive health effects underscores the asymptomatic nature of this translocation, and its preservation in the human population is likely due to random events.

Robertsonian translocations are a common chromosomal rearrangement involving the fusion of an entire acrocentric chromosome to another chromosome with the same centromere position. In humans, this type of rearrangement typically involves chromosomes 13, 14, 15, 21, and 22 [58]. These chromosomes are not canonical acrocentric chromosomes, as they lack a true centromere at the end of their short arm and instead have regions, the p12 band, where *ribosomal RNA* (*rRNA*) genes are located. These regions are hot spots for genomic rearrangements due to their high degree of sequence similarity [59,60]. Even the possible loss of some *rRNA* genes does not result in a detectable effect, as in this case, considering that a large portion of the rRNA genes in a cell is inactive [61,62].

The consequences of such rearrangements depend on the positions of the breakpoints on the involved chromosomes and can include various genetic outcomes, such as maintaining both centromeres, loss of *rRNA* genes, loss of a centromere, and so on. In the case described here, the centromeric satellites were retained in the rearranged chromosome, with one of the two centromeres (the cen21) appearing to be inactive based on the hybridization signals observed with the pZ21A probe (Figure 3), even if this has not been confirmed by CENP-C visualization. Moreover, the rDNA was not completely lost, as evidenced by specific in situ hybridization with an rDNA probe. Thus, we can suppose that the rearrangement became a meiotic problem that involved the rDNA regions of the two chromosomes 21 and 22, leading to the origin of the new t(21;22) chromosome. Previously, a Robertsonian translocation between chromosomes 14 and 15 was described in an individual with a karyotype containing 44 chromosomes, resulting in the formation of a new metacentric chromosome through the translocation [63]. However, the origin of the centromere present in this rearranged chromosome remains unclear.

### 4.3. Centromere Formation and Inactivation

During chromosome evolution, new centromeres have formed numerous times, and new syntenic groups have arisen in related species. Sometimes, chromosomes have varied their morphology by repositioning their loci, such as a chromosomal inversion comprising centromeres, but in other cases, they have retained the same sequence of loci, only repositioning the centromere by its activation/inactivation in different positions along the chromosome [20,64,65]. The emergence of these new centromeres during evolution is associated with the ability to develop new centromeres on a chromosomal region without adversely affecting chromosome functionality [66]. These silent occurrences of neocentromere formation have been documented in humans, indicating that they are not phenotypically detectable and are often identified incidentally [67,68].

In human cases, new centromeres that do not result in pathological phenotypes typically form in chromosomal regions that serve as true centromeres in other primate species. This suggests that certain chromosomal regions are highly predisposed to acquiring centromeric functions. Once formed, these new centromeres have a high probability of being retained within cells and populations, thus persisting at the evolutionary level [35,65,66,69].

In the rearranged t(21;22) chromosome, a new centromere did not emerge; instead, the centromeric sequences from both original chromosomes were detected, and over time, one of these satellite sequences may be gradually lost. However, as noted by Song et al. [63], newly formed karyotypes often fail to survive within a population of normal karyotypes, emphasizing the challenge of establishing new karyotypes without a mechanism to preserve these changes. This highlights the importance of conducting further studies on other members of the described family.

### 4.4. The Position Effect of Genes in the Nucleus

The position effect of genes within the nucleus suggests that a gene’s activity can be influenced independently of mutations directly affecting its sequence. In other words, for a gene to exhibit deregulation leading to a pathological phenotype, it may not necessarily require mutations; a change in its nuclear positioning alone could suffice. Recent evidence supports this notion, particularly in cases of leukemic cells featuring reciprocal translocations between chromosomes 7 and 12. In these instances, the *MNX1* gene, implicated in leukemia onset, remains unaltered in its sequence but experiences a shift in its nuclear position, resulting in its activation [30].

In our present case, the translocation occurs, presumably, at the level of the rDNA. Indeed, in situ hybridization with rDNA defined that these sequences are in the middle part of the rearranged chromosome, between the α satellite specific for chromosomes 21 and 22. The location of this chromosome in the inner part of the nucleus is coherent with this assumption; namely, the genes directly involved in the breakpoint do not modify their transcriptional properties.

Therefore, the difference between evolutionary chromosomal aberrations and those associated with chromosomal pathologies lies in the different intranuclear configuration of the centromere region: if the position does not change, the chromosomal alteration is silent and can thus be maintained in the population without problems in an “invisible” manner. If, instead, the position in the nucleus changes, the chromosomal aberration leads to the onset of genetic pathologies, resulting in the elimination of such aberration from the population.

### 4.5. Robertsonian Translocation and Chromosome Transmission

Robertsonian translocations are recognized as typical events for generating new chromosomes. The translocated t(21;22) chromosome was identified in five healthy individuals spanning three generations. Remarkably, this chromosome functions effectively within the cell, exhibiting no loss of vitality or reduction in fertility. Consequently, this new chromosomal configuration can persist for a prolonged period without any selective advantage or disadvantage. Its functionality is evident from the absence of negative impacts, such as abortion or other adverse events. This novel chromosome can be passed on to subsequent generations through a neutral mode of conservation, potentially leading to the formation of a karyotype with 44 chromosomes, as previously supposed considering similar data [63].

The presence of supernumerary dicentric chromosomes that are mitotically stable has already been described in numerous immortalized cell lines [70,71], but, to our knowledge, the one shown here appears to be the first case of a chromosome obtained by a translocation between chromosomes 21 and 22 that maintains both centromeres and is both mitotically and meiotically stable, transmitted through three generations without apparent phenotypic consequences. Other previously described cases of translocations between chromosomes 21 and 22 are instead associated with health problems and a high frequency of spontaneous miscarriages or Down syndrome in the analyzed families [72,73,74].

However, the formation of a new karyotype does not signify the emergence of a new species, as this chromosome configuration alone may not be adequate to induce post-zygotic isolation, a crucial step in the separation of new species via sympatric evolution. Indeed, rearrangements that do not cause sterility or post-zygotic incompatibility with other members of the population may only achieve evolutionary success by chance. In the postzygotic isolation of new animal species, differences in genes carry more significance than large structural chromosomal rearrangements [75], even if single chromosome mutations are potentially able to generate new species in one generation. Therefore, in a population, individuals with significant chromosomal rearrangements that do not lead to sterility or post-zygotic incompatibility with other members of the population may coexist alongside those with a standard "wild-type" karyotype, and these divergent karyotypes may only achieve evolutionary success through chance occurrences.

## 5. Conclusions

Chromosomes can undergo various types of rearrangements, either spontaneously or induced by external factors, leading to different outcomes. Depending on the nature of the rearrangement and the nuclear relocation of the chromosomal regions involved, the consequences for the cell can vary. Some rearrangements are asymptomatic, meaning they do not cause functional issues for the cell and can be passed on to offspring and spread in the population through random events. The family described here exemplifies such a case, where a chromosome is transmitted to offspring without apparent health problems for the carriers. This karyotype may already be spreading within the population, and the chance encounter between gametes carrying the t(21;22) chromosome can result in the formation of a zygote with 44 chromosomes. This configuration entails the disappearance of the acrocentric chromosomes 21 and 22 and the emergence of a metacentric chromosome formed by their fusion, thereby leading to the formation of a new karyotype structure in the human species within this population. It should be stressed that the casual identification of this type of chromosomal anomaly was possible by means of classical cytogenetic analysis, and it would not have been possible using molecular karyotyping methods such as array-CGH. This confirms that chromosomal banding analysis is still the best system for identifying balanced chromosomal abnormalities.

## Figures and Tables

**Table 1:** BAC probes used in FISH experiments.

BAC	Position ^(a)^	Start ^(b)^	End ^(b)^	Size ^(c)^
RP11-345K23	21q11.2	16,368,352	16,556,943	188,592
RP11-729O4	21q22.2	40,844,581	41,029,286	184,706
RP11-135B17	21q22.3	47,931,911	48,108,188	176,278
RP11-112D4	22q11.21	18,202,100	18,385,443	183,343
RP11-931F19	22q13.33	50,425,014	50,627,374	202,361
RP11-164E23	22q13.33	50,841,120	50,995,394	154,275
RP11-79O21	7p21.3	12,053,525	12,194,425	140,901
RP11-17H7	7q15.1	28,171,887	28,341,999	170,112
RP11-213E22	7q22.1	100,705,096	100,899,914	194,819

^(a)^ Chromosomal band; ^(b)^ distance in bp from the telomeric end of the short arm; ^(c)^ size in bp of the human DNA inserted in the BAC clone.

## Data Availability

All data are contained within the article.
